# One hundred years of the St Mark’s hospital polyposis registry

**DOI:** 10.1007/s10689-025-00463-x

**Published:** 2025-04-12

**Authors:** Vicky Cuthill, Andy Latchford, Sue Clark

**Affiliations:** 1 St Mark’s Hospital Polyposis Registry, St Mark’s Centre for Familial Intestinal Cancer, St Mark’s Hospital, London North West University Hospitals NHS Trust, London, UK; 2 Department of Surgery and Cancer, Imperial College, London, UK

**Keywords:** Familial adenomatous polyposis, Juvenile polyposis syndrome, Peutz-Jeghers syndrome, Registries, Data collection

## Abstract

The St Mark’s Hospital Polyposis Registry was founded in 1924, the first such unit in the world. This paper documents the development of the unit over the subsequent 100 years, which was inextricably linked to scientific and clinical advance in the field of polyposis syndromes.

## What is St Mark’s hospital?

St Mark’s Hospital in London was opened in 1835 by the surgeon Frederick Salmon as ‘The Infirmary for the Relief of the Poor afflicted with Fistula and other Diseases of the Rectum’ [1]. It grew rapidly, moving from the original small premises in Aldersgate Street to nearby Charterhouse Square, then to City Road. This new hospital first opened on 25th April 1854, St Mark’s day, becoming ‘St Mark’s Hospital for Fistula and other Diseases of the Rectum’. It remained there until 1995, when it moved to Northwick Park in Harrow, and later became one of the hospitals of the London North West University Hospitals NHS Trust (LNWUH). The hospital was the first to specialise entirely in colorectal disease.

## The early years– medical curiosity

The polyposis syndromes are rare; the commonest, familial adenomatous polyposis (FAP), was described by Harrison Cripps in 1882 [2] and several other independent descriptions followed.

**Fig. 1:**
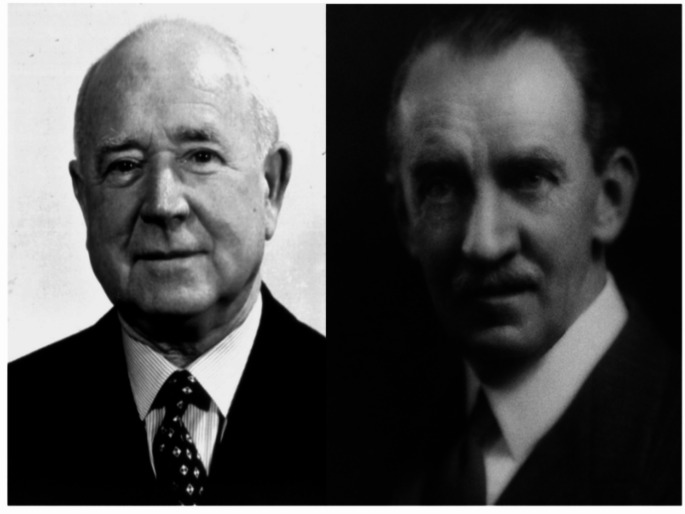
Cuthbert Dukes (left) and Percy Lockhart-Mummery (right)

By the early twentieth century St Mark’s had a burgeoning referral practice, and the concept of scientific medicine was gaining momentum. Percy Lockhart-Mummery (1875–1957) (Fig. 1) was a surgeon at St Mark’s at this time (on staff 1904-35), and had broad interests and imagination. His book ‘After Us’ [3] gives an insight into this era, and encompasses politics, town planning, economics and healthcare; he was surprisingly prescient on some subjects, including sexual equality, while other ideas were rather more far-fetched. He was clear, however, that ‘a better understanding…….will enable the doctor not only to treat his patient successfully, but to anticipate what is going to happen to prevent serious disease from supervening’ and that ‘ before medicine can become an exact science…it must resort to exact measurement’.

**Fig. 2:**
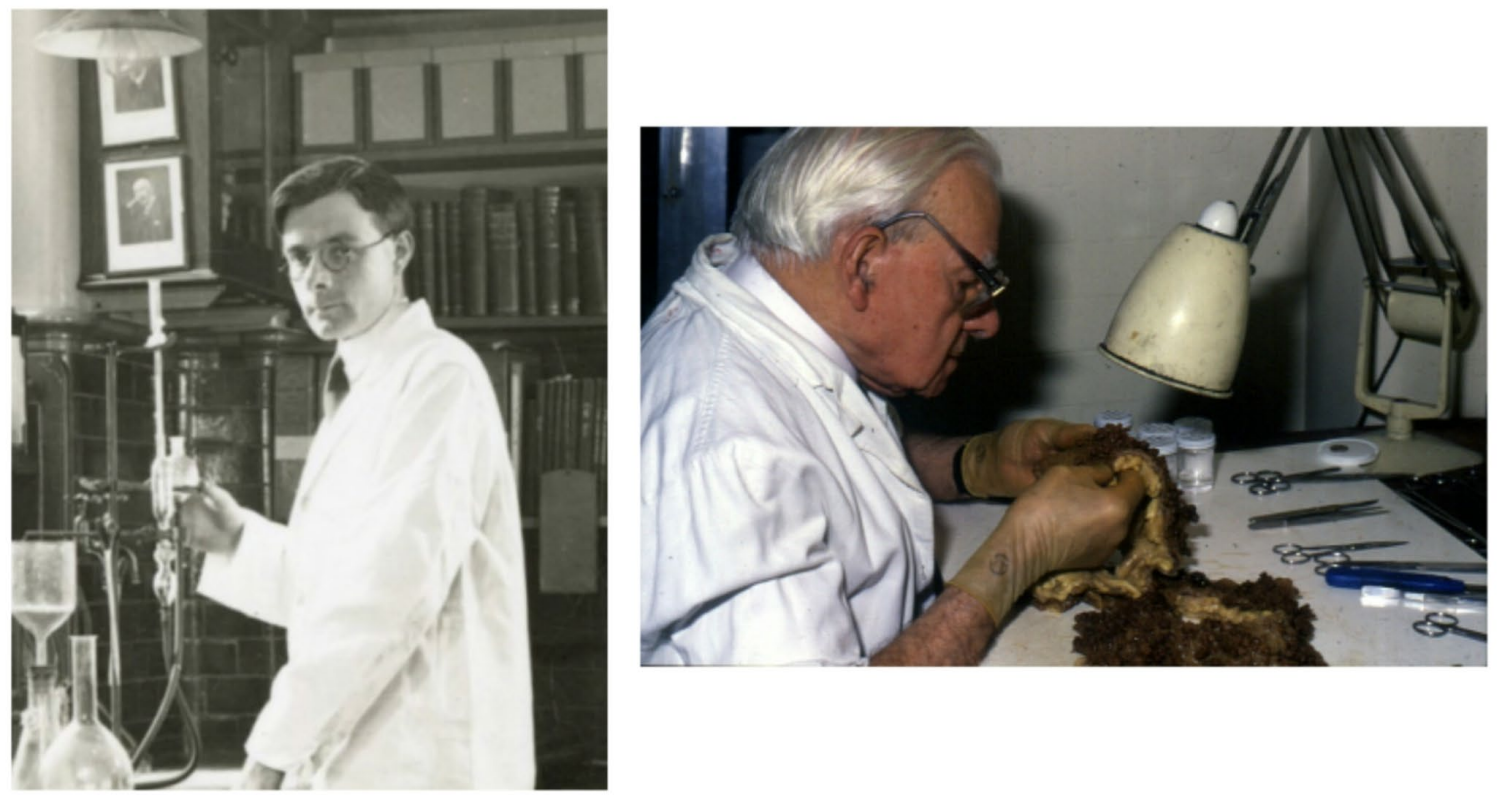
Dick Bussey in the pathology laboratory at St Mark’s c1924 and still at work cutting up polyposis specimens c1990

**Fig. 3:**
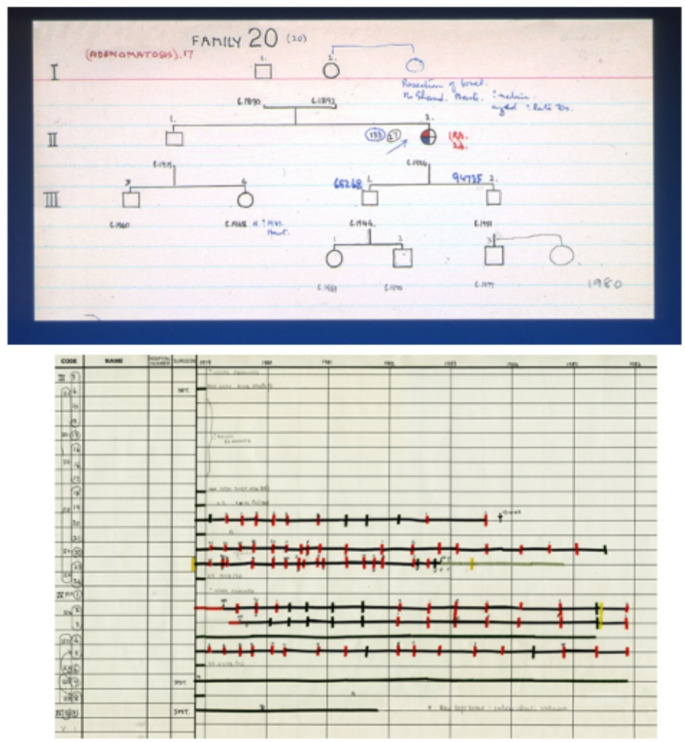
An original pedigree and longitudinal record of polyp progression

In 1922 the pathologist Cuthbert Dukes (1890–1971) (Fig. 1) joined St Mark’s, followed by the 17 year old laboratory assistant HJR (Dick) Bussey (1906–1993) (Fig. 2). Dukes had a particular interest in rectal cancer, and went on to create the eponymous staging system still widely used today. In 1924 he and Lockhart-Mummery started to document cases of patients with large numbers of polyps, and obtain family histories. At that time little could be done to help these patients. However, meticulous collection of records (Fig. 3) and pedigrees confirmed the dominant inheritance of FAP [4] and allowed what Dukes described as ‘confusing errors’ in previous reports [5] to be corrected. This included emphasising the need for histopathology to distinguish the adenomatous polyps of FAP from inflammatory and hamartomatous polyps and documenting progressive polyposis starting in adolescence with the almost inevitable occurrence of cancer at a young age. Although the first descriptions had all come from elsewhere, St Mark’s produced authoritative reviews backed by high quality data.

Most of the families identified had FAP, but some had Peutz-Jeghers (PJS) or juvenile polyposis syndrome (JPS). This culture of accurate data collection has provided the largest and most mature dataset worldwide, underpinning many of the advances in knowledge in this field, and continues in the unit to this day.

This work was also instrumental in developing an understanding of the development of colorectal cancer. In 1933 Lockhart-Mummery wrote a book on the subject [6] in which he argued that cancer was the result of genetic change in a normal cell which ‘ having previously been a good citizen, suddenly…commences to live independently of his fellow citizens to his own advantage and their detriment.’ He recognised that exposure to carcinogens (such as tar), in combination with inherited susceptibility, could result in mutation. In one of the many publications from around that time Lockhart-Mummery and Dukes postulated that FAP was the result of ‘an inherited instability of the epithelial cells of the large bowel which renders their nuclei particularly liable to undergo mutation’. Later Basil Morson (who succeeded Dukes as pathologist in 1956) described the ‘adenoma carcinoma sequence’ [7], with observations of patients with FAP providing some of the underlying evidence.

## Post-war– the ability to make a difference

The introduction of antibiotics and much lower risk general anaesthesia during the Second World War meant that bowel resection became safe enough to contemplate as a prophylactic procedure to prevent colorectal cancer in FAP. On 8th December 1948 a 24 year old woman underwent the first recorded prophylactic colectomy with ileorectal anastomosis (IRA), performed by the surgeon Oswald V Lloyd-Davies at St Mark’s; she went on to live into her 80’s. Other centres around the world (notably the Cleveland and Mayo Clinics in the USA) introduced a similar approach, which gradually became widespread.

It is perhaps worth remarking on the enormous advances that had been made, including the ability to diagnose FAP (using rigid sigmoidoscopy) from those at risk (identified by taking family histories), who were offered regular surveillance. Affected individuals could then be offered sphincter preserving prophylactic surgery (in many cases, though some still required panproctocolectomy and ileostomy if there was severe rectal polyposis) to prevent colorectal cancer. Flexible endoscopy did not exist, and the double-helix structure of DNA was not reported until 1953.

Overall, the work of the Polyposis Registry remained dominated by tracing families and characterising the polyposis syndromes. Morson published on the pathology and genetics of JPS; PJS featured in several papers produced from the unit. The World Health Organisation (which had recognised St Mark’s as a reference centre for classification of colorectal cancer and of pre-cancerous lesions) provided a grant 1968 to allow the Polyposis Registry to have a dedicated space.

## 1980’s and 1990’s– rapid change

There were three advances around this time that had a major impact on the care of patients with FAP and other polyposis syndromes. The first of these was the development of flexible endoscopy. It took some years for this to enter clinical practice, and advances in therapeutic techniques continue. It became possible to examine the entire large bowel, and after surgery to survey the rectum much more easily, and even to remove polyps.

In 1977 Sir Alan Parks and John Nicholls (consultant surgeons at St Mark’s 1959-82 and 1978–2006 respectively) pioneered the restorative proctocolectomy (RPC) allowing the entire large bowel to be removed without need for a permanentileostomy [8]. While this was originally developed for patients with ulcerative colitis, it rapidly became apparent that it would be of benefit to patients with FAP requiring removal of the rectum.

Finally, the breakthroughs of Sanger sequencing in 1977 and the polymerase chain reaction in 1983 allowed rapid advance in research in the role of genes in disease, including cancer.

**Fig. 4:**
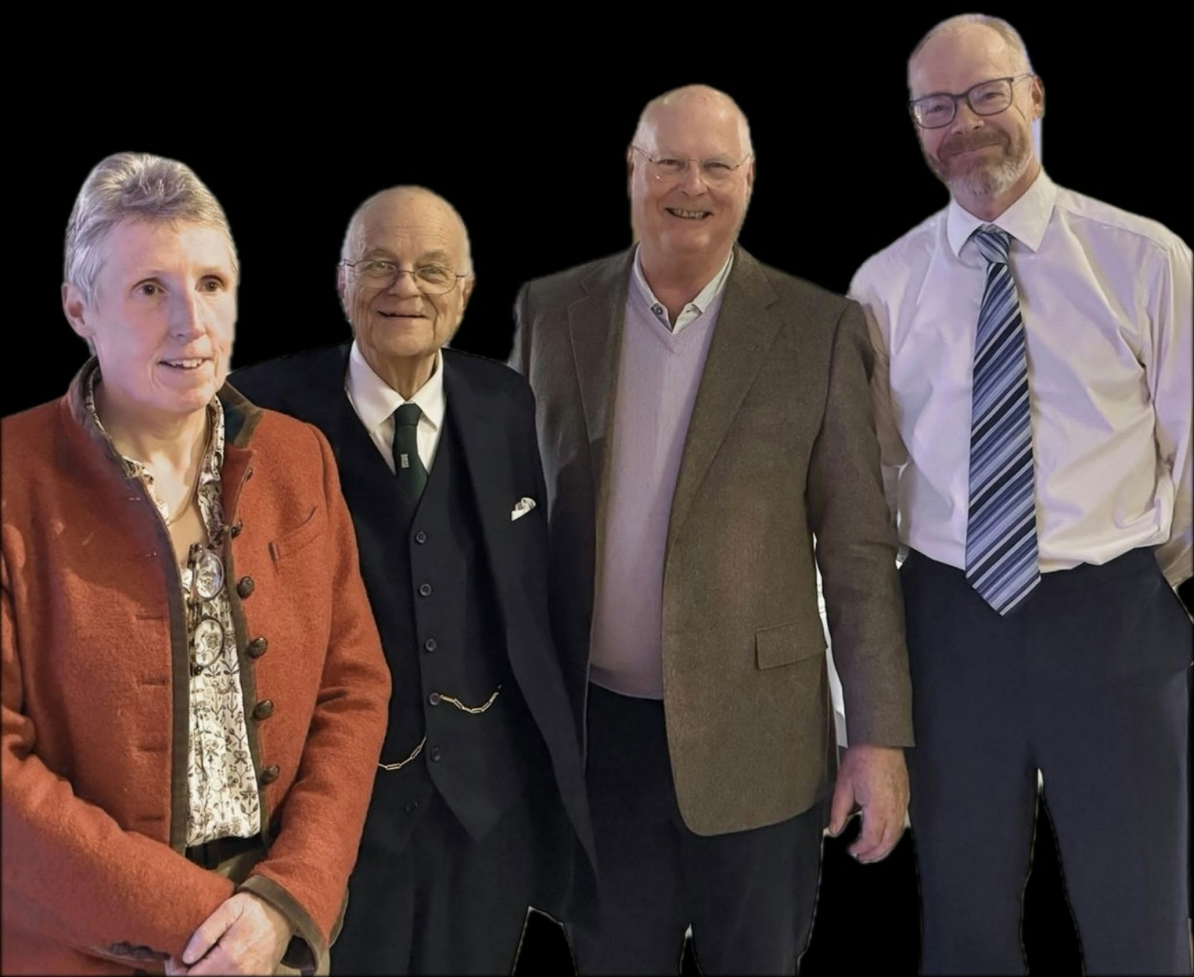
Directors of the St Mark’s Hospital Polyposis Registry at the Centenary dinner March 2024. Left to right: Sue Clark (2013-22), James Thomson (1985-92), Robin Phillips (1993–2012), Andy Latchford (2023-)

**Fig. 5:**
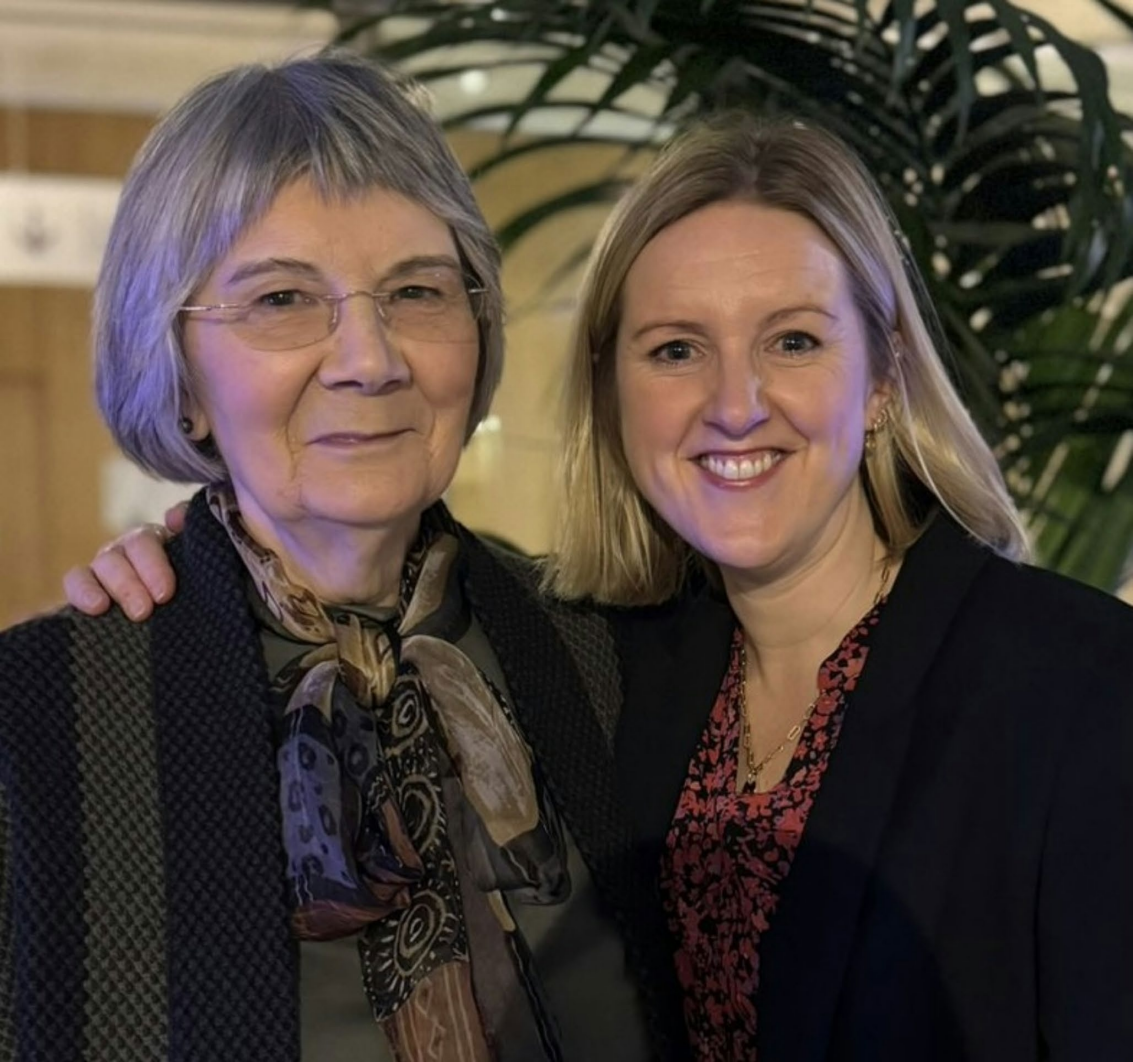
Kay Neale (left) and Vicky Cuthill (right) at the Centenary dinner

In 1985 the Polyposis Registry became a formal department at St Mark’s with the surgeon James Thomson as its first Director (Fig. 4); Bussey (who had gone on to obtain a PhD in 1970) was still in post (Fig. 2), alongside Sheila Ritchie, who was replaced by Judith Landgrebe in 1989. Kay Neale (Fig. 5), who had worked at St Mark’s since 1974, joined the Polyposis Registry staff 1984, where she remained until her retirement in 2016. This team was responsible for the gradual transition from the original ‘record card’ system to a computerised one.

By now there were about 300 patients with FAP regularly attending; there were records of more, with some families going back three or four generations, and accompanying clinical and pathological details. This era was ‘bookended’ by the publication of two definitive textbooks, the first in 1975 [9] and the second in 1994 [10].

Shortly after the establishment of flexible endoscopy in the UK, Robin Phillips (Fig. 4) became a surgeon at St Mark’s in the late 1980’s. His first research fellow was Allan Spigelman, whose research involved using flexible endoscopy to examine the stomach and duodenum of patients with FAP. He found that around 90% of the patients had duodenal adenomas, described their distribution and developed the Spigelman classification to facilitate documentation of the severity of duodenal adenomatosis [11].

Karen Nugent was the next research fellow; she focussed on outcomes in patients who had undergone prophylactic surgery since 1948. She showed that prophylactic IRA resulted in a very substantial improvement in life expectancy, but this still lagged behind the general population, the difference being due mainly to duodenal cancer, desmoid disease and cancer of the retained rectum [12]. She also showed that development of cancer in the retained rectum after IRA was a significant issue, something that the units at the Mayo and Cleveland Clinics reported at around the same time. The response to this varied. The Mayo Clinic and many European centres adopted RPC as the standard prophylactic procedure; the Cleveland Clinic, St Mark’s and many Scandinavian units developed a selective approach, using flexible colonoscopy (and later genotype) to assess the severity of polyposis, and recommending IRA in milder cases and RPC when the rectal polyp burden was higher.

In 1984 the Imperial Cancer Research Fund (ICRF), let by Walter Bodmer, established the Colorectal Cancer unit to undertake research at St Mark’s. His area of research centred around the role of genes in cancer, and Jeremy Jass, who had similar interests, had become a pathologist at St Mark’s. The Polyposis Registry could give them access to 124 members (affected and unaffected) of 13 families with FAP, and work on their DNA led to the identification of the site of the *APC* gene on chromosome 5 [13]. Interestingly his colleague Ellen Solomon, working in the same unit, studied DNA from about 40 sporadic colorectal cancers removed at St Mark’s, and found that the same region of chromosome 5 was missing in around a half of them; somatic loss of *APC* is of course now recognised as an early event in a majority of sporadic colorectal cancers. Leading on from these findings, details of the actual *APC* gene were published almost simultaneously by two groups in the US in 1991 [14, 15].

## International collaboration

In 1985 Sir Ian Todd, another surgeon at St Mark’s, 1985 convened a meeting Leeds Castle, Kent, of 30 professionals from across the world to discuss desmoid disease in FAP; they met again in 1987 and in 1989 became the Leeds Castle Polyposis Group. Biennial meetings continued, last in parallel with the International Collaborative Group on HPNCC, until the two group merged to form the International Society for Gastrointestinal Polyposis (InSiGHT) in 2003 [16].

## Translating advances into clinical care

Robin Phillips (Fig. 4) took over as Director in 1993, and set about improving the clinical services for patients by focussing research on the areas of greatest need and the way they were cared for. In 1995 St Mark’s moved from City Road to a newly renovated and relatively spacious building at Northwick Park. The co-located ICRF laboratories moved too, enabling the scientists (including Ian Frayling and Ian Tomlinson) and research fellows to continue close contact with the patients and clinicians, a stimulating and fruitful environment. At around the same time the ICRF Family Cancer Clinic was set up at St Mark’s to investigate families with a strong history of colorectal cancer, but who did not have polyposis; many of these turned out to have Lynch syndrome.

A string of research fellows (including Sue Clark and Andy Latchford) worked (largely fruitlessly!) on understanding the aetiology of desmoid and improving management. Chris Groves revisited Allan Spigelman’s original patients and documented the progression of duodenal disease [17], informing guidelines on surveillance intervals. Others were involved in various (also relatively fruitless) chemoprevention trials, and some preliminary work on gene therapy.

In 1996 genetic testing was made available (with the Polyposis Registry staff responsible for genetic counselling), allowing family members found not to be affected to avoid unnecessary colonoscopic surveillance. A much bigger endoscopy unit allowed flexible sigmoidoscopy to replace the usual practice of rigid sigmoidoscopy done in clinic when patients came for follow-up. This change, and increasing patient numbers, meant the system of ‘polyposis weeks’ had to end. During these weeks, the consultants devoted their clinics entirely to polyposis patients, and the registry staff attended; the waiting room provided an informal and much valued support group.

Spreading patients’ appointments throughout ‘normal clinics’ reduced exposure and supervision of the trainees seeing many of them, and made direct input from the Polyposis Registry more difficult. Many similar units around the world were staffed by genetic counsellors, but the approach at St Mark’s was different: the actual genetics is generally pretty straightforward, and diagnosis is usually not difficult; what patients really need is lifelong expert clinical care. So the National Health Service (NHS) was persuaded to fund a nurse endoscopist and increasing numbers of nurse practitioners, credentialed to deliver care. Patients were concentrated under the care of surgeons and gastroenterologists with expertise in polyposis syndromes, and a paediatric gastroenterologist, Warren Hyer, was recruited in 2003. In 2014 he was joined by Jackie Hawkins, the first Paediatric Nurse Practitioner. An annual patient information day was started, and the patients themselves formed a support group.

The registry database, initially a research tool, became increasingly important in patient care, flagging overdue surveillance, children needing genetic testing and so on. It moved from being a stand-alone system to the hospital’s servers, but continues to be funded entirely from charitable donations. The enlarging unit was managed by Kay Neale until her retirement in 2016, when Vicky Cuthill took over (Fig. 5).

Serrated polyposis syndrome patients were identified and added to the registry. The concept of on-table enteroscopy and ‘clean sweep’ to reduce the number of operations in PJS. When another group identified *SMAD4* pathogenic variants as a cause of JPS, the team at St Mark’s confirmed this in some, but not all cases [18]; they followed up by identifying *BPMR1A* mutation as the cause in most of the rest [19]. Repeated study of patients with a phenotype FAP, but no *APC* mutation identified, confirmed the existence of *MutYH*-associated polyposis [20] very shortly after the first description, and led to discovery of mutation *POLE* and *POLD1* in others [21]. Several publications have gone on to refine the resulting phenotypes.

Sue Clark returned to the unit as a consultant surgeon in 2006, and took over as Director in 2013; Andy Latchford came back as consultant gastroenterologist in 2013 (Fig. 4). They continued the programme of desmoid research (with Ashish Sinha, now a consultant surgeon at St Mark’s, previously as one of the research fellows), and also started ongoing work on the process of carcinogenesis in the duodenum and ileoanal pouch.

## Covid-19 and after

The Covid-19 pandemic saw staff redeployed to intensive care and wards, and elective endoscopy and surgery paused. St Mark’s Hospital itself struggled to provide complex services to patients from around the UK in a hospital overwhelmed by local emergency cases. In 2021 St Mark’s Hospital moved most of its services from Northwick Park to the Central Middlesex site, also part of LNWUH. This allowed the St Mark’s Hospital Polyposis Registry and Family Cancer Clinic to move into the same large space, facilitating their merger into the St Mark’s Centre for Familial Intestinal Cancer (SMCFIC), with staff now covering both Lynch and the polyposis syndromes, increasing resilience and efficiency. The following year Vicky Cuthill became a Nurse Consultant, and in 2023 Andy Latchford became Director (and co-Director of SMCFIC, with Kevin Monahan, lead of the Family Cancer and Lynch service).

### A National network

Building evidence that patients with rare diseases are best cared for in specialist units led the NHS Strategy for Rare Diseases to include the concept of Rare Disease Collaborative Networks; these consist of centres in which a multidisciplinary team provides high quality specialist care, with centres using the same guidelines, taking part in regular network meetings and collaborating in research. St Mark’s provided care for nearly 2000 polyposis patients from around the UK (probably the biggest single provider of ongoing care worldwide), while others were looked after at other units, which mainly relied on the enthusiasm of dedicated individual clinicians, or received often suboptimal care at their local hospitals. In 2021 Sue Clark successfully applied to the NHS to set up a network for Hereditary Gastrointestinal Polyposis Syndromes. The centres are in Edinburgh, Cardiff, Manchester/Liverpool, Birmingham, Southampton, Exeter/Plymouth and St Mark’s, and the aim is that all patients in the UK will eventually be cared for at or under the direction of their closest centre [22].

## Conclusion

**Fig. 6:**
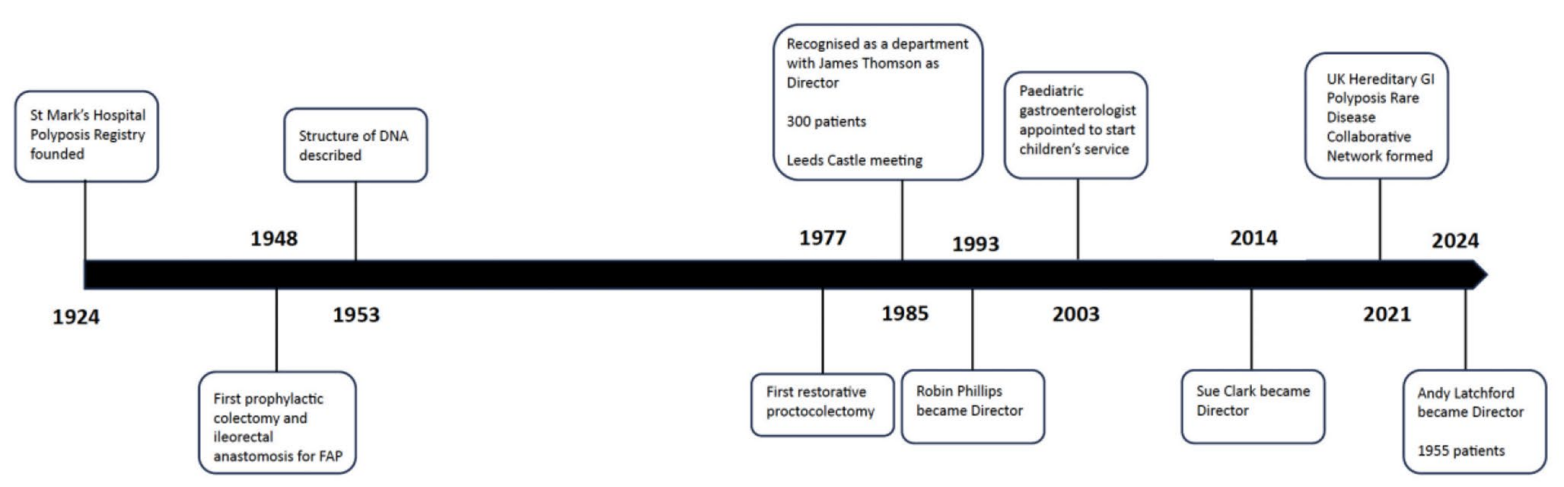
Timeline of the St Mark’s Hospital Polyposis Registry

The enthusiasm of a small number of individuals drove the evolution of the St Mark’s Hospital Polyposis Registry from an early exercise in curiosity-driven data collection, to a unit that could actually develop and provide effective clinical care for patients with the rare polyposis syndromes (Fig. 6). The meticulously collected dataset and access to the patients has provided a valuable resource for laboratory and clinics studies; the location within a small specialist hospital at the forefront of research into colorectal cancer development and innovation in diagnostics and surgery facilitated both these aspects. The drive to improve care of patients worldwide, and broaden research collaboration, led directly to the foundation of InSiGHT and the beginnings of a rational national polyposis service in the UK.

## Data Availability

No datasets were generated or analysed during the current study.
